# Intra- and Inter-scanner Reliability of Scaled Subprofile Model of Principal Component Analysis on ALFF in Resting-State fMRI Under Eyes Open and Closed Conditions

**DOI:** 10.3389/fnins.2018.00311

**Published:** 2018-05-25

**Authors:** Li-Xia Yuan, Jian-Bao Wang, Na Zhao, Yuan-Yuan Li, Yilong Ma, Dong-Qiang Liu, Hong-Jian He, Jian-Hui Zhong, Yu-Feng Zang

**Affiliations:** ^1^Key Laboratory for Biomedical Engineering of Ministry of Education, Center for Brain Imaging Science and Technology, College of Biomedical Engineering and Instrumental Science, Zhejiang University, Hangzhou, China; ^2^Center for Cognition and Brain Disorders and the Affiliated Hospital, Hangzhou Normal University, Hangzhou, China; ^3^Zhejiang Key Laboratory for Research in Assessment of Cognitive Impairments, Hangzhou, China; ^4^Institutes of Psychological Sciences, College of Education, Hangzhou Normal University, Hangzhou, China; ^5^Center for Neurosciences, The Feinstein Institute for Medical Research, Manhasset, NY, United States; ^6^Research Center of Brain and Cognitive Neuroscience, Liaoning Normal University, Dalian, China

**Keywords:** principal component analysis, scaled subprofile model, intra-scanner reliability, inter-scanner reliability, resting-state fMRI

## Abstract

Scaled Subprofile Model of Principal Component Analysis (SSM-PCA) is a multivariate statistical method and has been widely used in Positron Emission Tomography (PET). Recently, SSM-PCA has been applied to discriminate patients with Parkinson's disease and healthy controls with Amplitude of Low Frequency Fluctuation (ALFF) from Resting-State Functional Magnetic Resonance Imaging (RS-fMRI). As RS-fMRI scans are more readily available than PET scans, it is important to investigate the intra- and inter-scanner reliability of SSM-PCA in RS-fMRI. A RS-fMRI dataset with Eyes Open (EO) and Eyes Closed (EC) conditions was obtained in 21 healthy subjects (21.8 ± 1.8 years old, 11 females) on 3 visits (V1, V2, and V3), with V1 and V2 (mean interval of 14 days apart) on one scanner and V3 (about 8 months from V2) on a different scanner. To simulate between-group analysis in conventional SSM-PCA studies, 21 subjects were randomly divided into two groups, i.e., EC-EO group (EC ALFF map minus EO ALFF map, *n* = 11) and EO-EC group (*n* = 10). A series of covariance patterns and their expressions were derived for each visit. Only the expression of the first pattern showed significant differences between the two groups for all the visits (*p* = 0.012, 0.0044, and 0.00062 for V1, V2, and V3, respectively). This pattern, referred to as EOEC-pattern, mainly involved the sensorimotor cortex, superior temporal gyrus, frontal pole, and visual cortex. EOEC-pattern's expression showed fair intra-scanner reliability (ICC = 0.49) and good inter-scanner reliability (ICC = 0.65 for V1 vs. V2 and ICC = 0.66 for V2 vs. V3). While the EOEC-pattern was similar with the pattern of conventional unpaired *T*-test map, the two patterns also showed method-specific regions, indicating that SSM-PCA and conventional *T*-test are complementary for neuroimaging studies.

## Introduction

Identification of reproducible and region-specific effects that characterize normal or diseased brain state is one of the most important goals of brain functional imaging studies. The Scaled Subprofile Model of Principal Component Analysis (SSM-PCA) is one of the earliest multivariate data analytic techniques that are available to recognize significant group-dependent and region-specific effects (Moeller et al., 1987; Moeller and Strother, 1991; Alexander and Moeller, 1994; Eidelberg, 2009). The SSM-PCA is one form of regional covariance analysis, identifying functional interaction patterns among brain regions that are spatially distributed throughout the brain (Moeller et al., 1987). Commonly, the SSM-PCA has been applied to differentiate two groups of subjects (e.g., patients vs. healthy controls) (Alexander and Moeller, 1994; Spetsieris and Eidelberg, 2011; Wu et al., 2013; Tomše et al., 2017). The brain images of the two groups are decomposed to be a linear combination of a series of spatial patterns (i.e., images) by SSM-PCA. Each pattern is expressed in each subject with a Subject Scaling Factor (SSF), which can be prospectively assessed and compared between groups and validated with disease severity and neuropsychological test scores (Alexander and Moeller, 1994; Eidelberg, 2009).

SSM-PCA was first proposed to analyze data from Positron Emission Tomography (PET) (Moeller et al., 1987), and had been widely applied to investigate the effects of neurological and psychiatric illness on brain function, such as Alzheimer's disease (Alexander and Moeller, 1994), Parkinson's disease (Eidelberg et al., 1995), major depressive disorder (Sackeim et al., 1993), acquired immune deficiency syndrome dementia complex (Rottenberg et al., 1987), neoplastic disease (Anderson et al., 1988), and normal aging (Pagani et al., 2016). SSM-PCA was then utilized to deal with structural, perfusion, and diffusion Magnetic Resonance Imaging (MRI) metrics, including white and gray matter density (Brickman et al., 2007, 2008; Bergfield et al., 2010), gray matter volume (Guo et al., 2014; Steffener et al., 2016), cerebral blood flow (CBF) *(*Asllani et al., 2008; Teune et al., 2014), and fractional anisotropy (Gazes et al., 2016). More recently, SSM-PCA was applied to investigate Parkinson's disease-related covariance brain pattern with a Resting-State Functional MRI (RS-fMRI) metric (Wu et al., 2015), namely Amplitude of Low Frequency Fluctuation (ALFF) (Zang et al., 2007), revealing that the subject's expression of this pattern is capable of discriminating patients from healthy volunteers. RS-fMRI has many metrics and thousands of papers have been published on various brain disorders, however to our best knowledge, only one has utilized SSM-PCA (Wu et al., 2015).

Reliability is the cornerstone of any scientific measurement (Bennett and Miller, 2010). The intra- and inter-scanner reliability is an important metric for quantification of fMRI measurement reliability, given increasing research interest relying on the ability to combine the data from multiple scanners into larger, integrative data sets. For SSM-PCA, we found that only one study measured the test-retest (i.e., intra-scanner) reliability with PET images from two groups of subjects (Ma et al., 2007). That study demonstrated the very high test-retest reliability of the SSF analysis. No study has investigated inter-scanner reliability. The current studies investigated both intra- and inter-scanner reliability with the following 3 considerations.

First, we simulated a between-group design, i.e., comparison between two groups. SSM-PCA has been widely used to compare two groups of subjects, e.g., patient group vs. healthy group. The reliability of SSM-PCA is different from the reliability of other metrics. For example, the test-retest reliability of ALFF in RS-fMRI is usually tested in a single group of healthy subjects (Zou et al., 2015b), and it is relatively easy to scan a group of healthy subjects twice. But SSM-PCA should be performed on the between-group design to gain the reliability of the pattern as well as its expression for each subject, i.e., SSF. It is rather difficult to scan both the patient group and healthy group twice, especially in two different scanners. Moreover, the brain activity in the patients usually changes more than that in the healthy group over time, which affects the intra- and inter-scanner reliability. Therefore, the current study simulated two groups of subjects with a single group of healthy subjects to investigate both the intra-scanner (i.e., test-retest) reliability and the inter-scanner reliability.

Second, we used a RS-fMRI dataset under Eyes Open (EO) and Eyes Closed (EC) conditions. In RS-fMRI, EO and EC states are two resting physiological states with distinct differences in a few brain regions, and more importantly, these differences are highly reproducible across studies (Yang et al., 2007; Yan et al., 2009; Liu et al., 2013; Yuan et al., 2014; Zou et al., 2015a). EO and EC are usually for within-group designs. By randomly dividing one group into two subgroups and performing subtraction between conditions, e.g., EC-EO group and EO-EC group, between-group designs can be imitated with within-group data.

Third, we compared the spatial patterns generated by the multivariate method of SSM-PCA with that by the univariate statistical method of voxel-wise *T*-test. With a proper threshold, the surviving brain voxels of a *T* map implies the existence of significant difference between two groups (or conditions). By contrast, the surviving brain voxels of a SSM-PCA map mean that these voxels contribute more than other voxels to the difference between groups. We therefore were interested in studying the similarities and differences between the *T* map and SSM-PCA pattern with a certain threshold.

## Materials and methods

### Subjects

The experiment was approved by the ethics committee at the Center for Cognition and Brain Disorders, Hangzhou Normal University (HZNU). Signed informed consent was obtained from all subjects prior to data acquisition. Twenty-one healthy subjects (21.8 ± 1.8 years old, 11 females) participated in all 3 visits of MRI scans. All subjects were prescreened with a telephone questionnaire to exclude history of neurological illness or psychiatric disorders.

### Data acquisition

RS-fMRI dataset was obtained on 3 visits (V1, V2, and V3), with V1 and V2 (separated by 14 ± 1 days) on a scanner and V3 (230 ± 8 days from V2) on a different scanner. For each visit, participants underwent two RS-fMRI scans, during which they were asked to relax with either EC or EO. The order of the two acquisitions was counter-balanced across subjects.

MR images of V1 and V2 were obtained on a GE 3T scanner (MR-750, GE Medical Systems, Milwaukee, WI) with an eight-channel head coil at the Center for Cognition and Brain Disorders of HZNU. To minimize the head movement, subjects laid supine with their heads snugly fixed by straps and foam pads. The Blood-Oxygenation-Level-Dependent (BOLD) images were acquired using a gradient echo Echo-Planar Imaging (EPI) pulse sequence with the following parameters: Repetition Time (TR)/Echo Time (TE) = 2,000/30 ms, Flip Angle (FA) = 60°, 43 slices with interleaved acquisition, thickness/gap = 3.4/0 mm, Field Of View (FOV) = 220 × 220 mm^2^ with an in-plane resolution of 3.44 × 3.44 mm^2^. The duration of each resting-state scan was 8 min. A high-resolution 3D volume imaging was performed with a spoiled gradient-recalled pulse sequence (176 sagittal slices, thickness = 1 mm, TR/TE = 8.1/3.1 ms, FA = 9°, FOV = 250 × 250 mm^2^).

Data of V3 were acquired on a Siemens 3T scanner (Prisma, Siemens Healthineers, Erlangen, Germany) at the Center for Brain Imaging Science and Technology of Zhejiang University (ZJU). The BOLD EPI sequence parameters were the same as those on the GE scanner except FA = 90°. The 3D T1-weighted images were acquired with a Magnetization-Prepared Rapid-Acquisition Gradient Echo (MPRAGE) sequence (176 sagittal slices, thickness = 1 mm, TR/TE = 1,800/2.28 ms, inversion time = 755 ms, FA = 8°, echo spacing = 7.1 ms, turbo factor = 208, FOV = 250 × 250 mm^2^).

### Data preprocessing

Functional MRI data were preprocessed with Resting-State fMRI Data Analysis Toolkit plus V1.2 (RESTplus V1.2, http://restfmri.net/forum/index.php). Preprocessing procedures included removal of the first 10 frames, slice-timing correction, realignment to the first image for motion correction, coregistration of individual averaged functional images to T1 images, and spatially normalization into the standard Montreal Neurological Institute (MNI) brain space using the deformation field from segmentation of T1 images. All images were then resampled into 3 × 3 × 3 mm^3^ voxels, and smoothed using an isotropic Gaussian filter with a Full Width at Half Maximum (FWHM) of 6 mm. For all the subjects, the maximum translation and rotation were less than 1.5 mm and 1.5°, respectively. After removing the linear drift, ALFF was calculated based on the same procedures reported previously (Zang et al., 2007) with RESTplus. Briefly, the time courses of RS-fMRI signal were first converted to frequency domain with the Fast Fourier Transform (FFT). Then, the averaged amplitude across a frequency band of 0.01–0.08 Hz yielded ALFF. For each subject, the ALFF map was divided by the global mean ALFF value within a whole brain mask in RESTplus.

### SSM-PCA analysis

In most previous applications of SSM-PCA, image data from both patients and healthy controls were put together for analysis, and then a disease-related spatial covariance pattern was identified if significant difference in a pattern's expression was found between the patients and healthy controls by two-sample *T*-test (Alexander and Moeller, 1994; Spetsieris and Eidelberg, 2011; Tomše et al., 2017). The current study was a within-group design, i.e., comparison between two conditions within the same group of subjects, which is also useful for longitudinal follow-up or intervention studies in clinical research. To imitate the analytic procedure in most existing SSM-PCA studies, 21 subjects in this study were randomly divided into two groups with matched age and gender, i.e., EC-EO group (*n* = 11) and EO-EC group (*n* = 10). In detail, for the EO-EC group, we subtracted ALFF map of EC from ALFF map of EO for each subject to generate the difference map, and vice versa for the EC-EO group.

Based on a modified PCA, SSM-PCA decomposes the metric (ALFF in the current study) maps from all the subjects (EC-EO group and EO-EC group here) into a linear combination of orthogonal components. Each component is a whole-brain image, usually named as a “pattern.” Each voxel's value of any component is a weight representing the contribution of that voxel to the corresponding pattern. Voxels with a relatively large weight in the pattern was called “network” in Spetsieris and colleagues' paper on metabolic PET (Spetsieris et al., 2015). The pattern was also termed as Group Invariant Subprofile (GIS) (Moeller and Strother, 1991; Alexander and Moeller, 1994). The projection of each individual's ALFF map onto a pattern is regarded as the pattern's expression in the subject, which is also called Subject Scaling Factor (SSF). The SSFs are then used in further group-level statistical analysis, e.g., *T*-test between two groups or correlation with behavioral variables.

The mathematical basis for SSM-PCA has been previously described in detail (Moeller and Strother, 1991; Alexander and Moeller, 1994; Spetsieris and Eidelberg, 2011). Briefly, the difference ALFF maps were arranged first into an *M* × *N* dimensional data matrix, where each column represents all the voxels from each subject. *M* is the number of voxels and *N* is the number of subjects. Secondly, we centered each column to zero, and then acquired the Group Mean Profile (GMP) as the mean value of each row. Thirdly, the data matrix of each row was centered to zero with GMP to obtain the Subject Residual Profile (SRP). As in regular PCA, the reduced Singular Value Decomposition (SVD) was utilized to factorize SRP (Jolliffe, 2002; Spetsieris and Eidelberg, 2011):

(1) $$\begin{array}{r} U\Sigma V^{T}=SVD\left(SRP\right), \end{array}$$

where ***U*** is a *M* × *N* matrix composed of the left unit-normalized orthogonal singular vectors as columns, Σ is a *N* × *N* diagonal matrix composed of singular values σ_*k*_, where *k* is the component number, and ***V*** is a *N* × *N* matrix composed of the right unit-normalized orthogonal singular vectors as columns. Then, the GISs (namely patterns) and SSFs (namely patterns' expressions in each subject) can be computed as follows:

(2) $$\begin{array}{r} GIS_{ik}=U_{ik}, \end{array}$$

(3) $$\begin{array}{r} SSF_{jk}=\sum_{i\text{ = }1}^{M}\left(SRP_{ij}\times GIS_{ik}\right) \end{array}$$

in which *i* is the voxel number and *j* is the subject number. Variance Accounting For (VAF) represents the ratio of variance corresponding to every GIS to the total variance, calculated by:

(4) $$\begin{array}{r} VAF_{k}=\sigma_{k}^{2}/\sum_{k=1}^{N}\sigma_{k}^{2}. \end{array}$$

Two-sample *T*-test was then performed on SSFs to assess their difference between the EC-EO group and EO-EC group. GIS, whose SSF with *p* < 0.05, was considered to be the EC and EO difference-related spatial covariance pattern (hereafter named as EOEC-pattern). The GISs and SSFs were derived based on the SSMPCA toolbox (http://www.feinsteinneuroscience.org).

### Intra-scanner and inter-scanner reliability analysis

The intra-scanner and inter-scanner reliability of SSF from SSM-PCA was measured with Intra-Class Correlation (ICC). ICC for each pair of metrics from two visits was calculated as below (Shrout and Fleiss, 1979):

(5) $$\begin{array}{r} ICC=\left(BMS-WMS\right)/\left(BMS+WMS\right), \end{array}$$

where BMS and WMS are the mean squares values of between-target and within-target SSFs. To illustrate the similarity between EOEC-patterns and their SSFs between each pair of MRI scans, Pearson correlation coefficient (*r*) was also calculated (Zang et al., 2017). The effect of group division on the ICC of SSF was investigated by repeating the SSM-PCA with random division of subjects for 1,000 times with bootstrapping.

### EOEC-pattern generalization across visits

To investigate the generalization of EOEC-pattern across serial MRI datasets, we used Topographic Profile Rating (TPR) algorithm (Eidelberg et al., 1995; Ma et al., 2007). TPR quantifies the expression of a given pattern in an individual subject by the inner product of the pattern and the individual subject's SRP. The individual subject's SRP is acquired by using the GMP image associated with the derivation of the original pattern. For example, an EOEC-pattern was obtained from V1 data and then projected onto V2 and V3 data to compute SSFs. Two-sample *T*-test was performed to compare between the EC-EO group and EO-EC group for V2 and V3, respectively. ICC of V2 against V3 was also calculated as an additional way for measuring reliability, as was done by Ma and colleagues (Ma et al., 2007). Similarly, the EOEC-pattern from V2 or V3 was projected onto the data in the other two visits, and intra- or inter-scanner reliability was measured, in addition to the other indicators of reliability described in section Intra-Scanner and Inter-Scanner Reliability Analysis above.

### Comparison between EOEC-pattern and univariate statistical *T* map

In order to compare the EOEC-patterns from SSM-PCA and the *T* maps from univariate statistics, Dice Similarity Coefficient (DSC) was utilized. Univariate two-sample *T*-test was performed between EC-EO group and EO-EC group. A corrected *p* < 0.05 was used with AlphaSim in software RESTplus V1.2. This corrected *p* value corresponded to a combined threshold of single voxel *p* < 0.05 and cluster sizes larger than a certain number of voxels, which was determined on an estimated smoothing kernel size (Full Width at Half Maximum (FWHM) listed in Table 1) according to their *T* maps. It should be noted that there is currently no widely accepted method to determine the threshold for SSM-PCA pattern maps. To render it more comparable with the *T* map, we used the same cluster size threshold (Table 1) for the *z*-transformed EOEC-pattern map, sorted the absolute *z* value, and then determined the |*z|* threshold (Table 1), by which the total number of voxels of EOEC-patterns were kept almost the same as that of *T* maps (Table 1). DSC was computed as below (Rombouts et al., 1997):

(6) $$\begin{array}{r} DSC=2|A\cap B|/\left(\left|A|+|B\right|\right), \end{array}$$

where|*A*|, |*B*|, and |*A* ∩ *B*| are the total voxel numbers of the EOEC-pattern, *T* map, and their overlap, respectively.

**Table 1:** Parameters used in calculating DSC for comparison between EOEC-pattern and univariate statistical *T* map.

	**V1**	**V2**	**V3**
FWHM (mm) for *T* map	[12.1 12.9 11.3]	[11.9 12.6 11.2]	[13.0 13.7 13.6]
Cluster size threshold (voxel)	915	952	1137
Voxel number in *T* map	7669	10246	14913
|*z*| threshold for EOEC-pattern	1.40	1.27	1.01
Voxel number in EOEC-pattern	7668	10246	14911

*DSC, Dice Similarity Coefficient; FWHM, Full Width at Half Maximum*.

## Results

### EOEC-pattern identification

**Figure 1:**
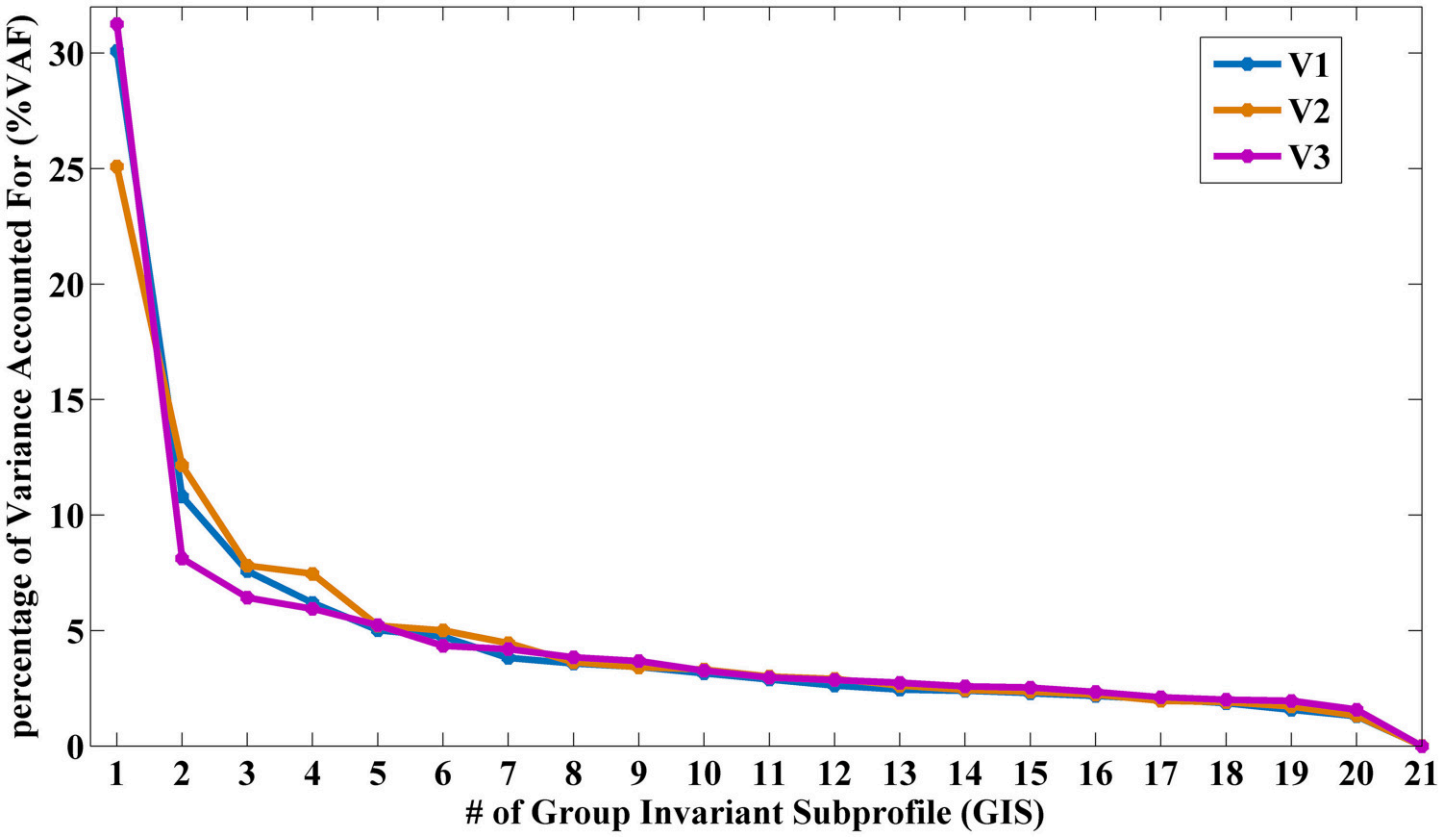
The percentage of VAF (%VAF) for each GIS in V1, V2, and V3. (VAF, Variance Accounting For; GIS, Group Invariant Subprofile).

**Figure 2:**
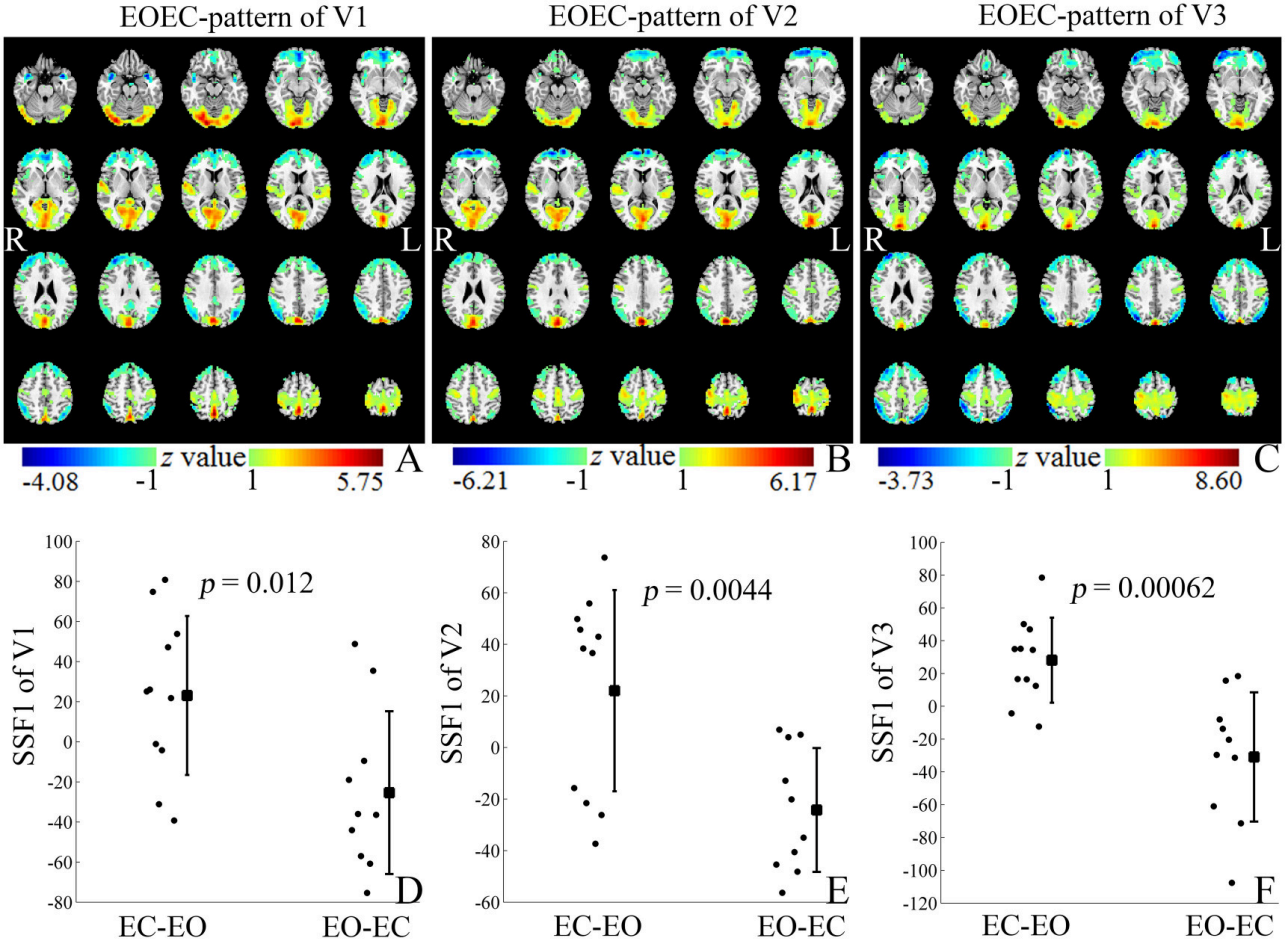
The *z*-transformed EOEC-patterns (with |*z*| > 1) **(A–C)** and their SSFs **(D–F)** of datasets V1–3. Positive and negative z values represented higher and lower ALFF in EC than EO, respectively. The *z* coordinates of each slice were from −25 to 70 mm with slice spacing of 5 mm (SSF, Subject Scaling Factor).

As shown in Figure 1 and Supplementary Table 1, the VAF of GIS1 for V1, V2, and V3 was remarkably larger than that of GIS2. Only the SSF of GIS1 showed significant difference (*p* < 0.05) between the EC-EO group and EO-EC group for each visit. Thus, GIS1 was named as the EOEC-pattern map for V1–3. As the VAF for GIS21 was zero, GIS21 and its SSF were ignored in *T*-test and reliability analysis. Figures 2A–C displayed the topography of *z*-transformed EOEC-patterns with a threshold of |*z*| > 1 and their SSFs in V1–3. Combining the SSF distribution and EOEC-pattern, positive *z* represented higher ALFF in EC than EO, mainly including the visual cortex, temporal cortex, and sensorimotor cortex. Inversely, negative *z* represented lower ALFF in EC than EO, mainly involving frontal pole and posterior parietal cortex. For each of these patterns, SSF values were significantly elevated in the EC-EO group compared to the EO-EC group (Figures 2D–F).

### Intra- and inter-scanner reliability of EOEC-pattern and its expression

#### Reliability by ICC

**Figure 3:**
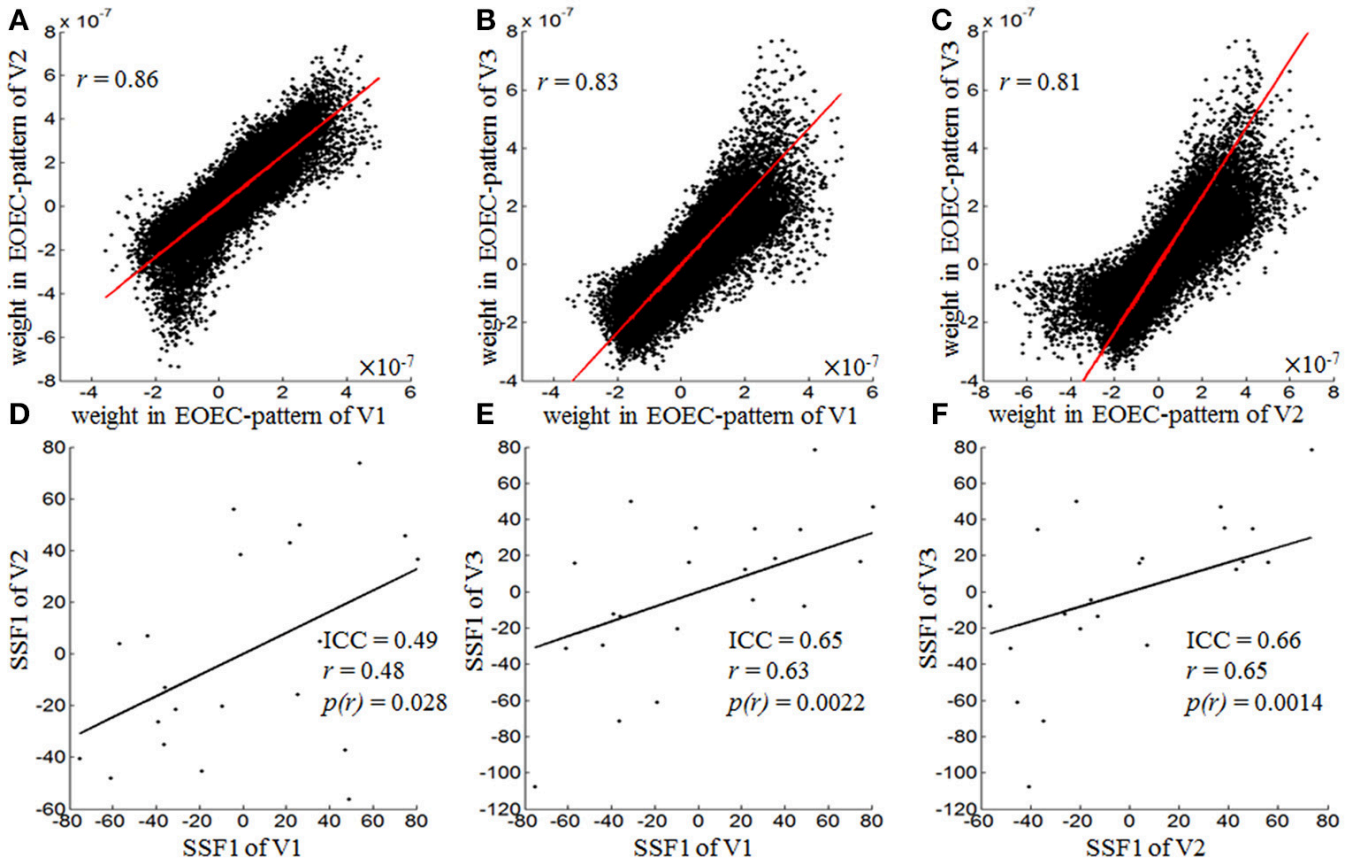
Pearson correlation of EOEC-patterns **(A–C)** and reliability of their expressions **(D–F)**, i.e., SSF1, of each pair of visits. The Pearson correlation coefficients for EOEC-patterns of V1 vs. V2 (intra-scanner), V1 vs. V3 (inter-scanner), and V2 vs. V3 (inter-scanner) were 0.86, 0.83, and 0.81, respectively, and the ICCs for EOEC-pattern expressions of V1 vs. V2, V1 vs. V3, and V2 vs. V3 were 0.49, 0.65, and 0.66, respectively (SSF1, Subject Scaling Factor of GIS1, namely EOEC-pattern here; ICC, Intra-Class Correlation; *r*, Pearson Correlation Coefficient).

As shown in Figure 3, the PCC between both intra- and inter-scanner EOEC-patterns was high (above 0.8, *p* < 0.001) (Figures 3A–C). The intra-scanner reliability of EOEC-patterns' expressions, i.e., SSFs, was fair (0.4~0.59) (Cicchetti, 1994). Interestingly, the inter-scanner reliability of SSF was good (0.6~0.74) (Figures 3D–F; Cicchetti, 1994). The PCC between each pair of visits was very similar with ICC. Supplementary Table 2 listed the intra- and inter-scanner ICCs of all the SSFs corresponding to GIS1-20. Except for ICC of SSF1, all ICCs of SSF2-20 were smaller than 0.4. The mean value, standard deviation, and 95% confidence interval of ICC of EOEC-patterns' expressions from bootstrapping were listed in Table 2. The table demonstrated that the ICC variation is very small compared with the mean value.

**Table 2:** ICC results of EOEC-pattern's expression from the random group selection for 1,000 times with bootstrapping.

		**V1 vs. V2**	**V1 vs. V3**	**V2 vs. V3**
EOEC-pattern's expression	Mean± std	0.48± 0.04	0.61± 0.03	0.66± 0.03
	95% confidence interval	[0.43 0.55]	[0.57 0.66]	[0.62 0.67]

*ICC, Intra-Class Correlation*.

#### EOEC-pattern generalization across visits

We calculated the expression (i.e., SSF) of each EOEC-pattern (V1, V2, and V3) on the other two datasets. As shown in Table 3, the ICCs between expressions of a given EOEC-pattern in the other two datasets was approximate to those between each pair of SSFs obtained from the EOEC-patterns of their own visits (Figures 3D–F). Two-sample *T*-test showed excellent cross-validation results (Table 3).

**Table 3:** EOEC-pattern generalization results across visits.

	**Expression of** **EOEC-pattern of** **V1**		**Expression of** **EOEC-pattern of** **V2**		**Expression of** **EOEC-pattern of** **V3**	
	**V2**	**V3**	**V1**	**V3**	**V1**	**V2**
*p-*value	0.0082	0.00099	0.0072	0.00038	0.0084	0.0054
*T*-value	2.95	3.89	3.01	4.31	2.94	3.14
Cohen *d*	1.09	1.30	1.11	1.37	1.09	1.14
ICC	0.64		0.71		0.46	

*ICC, Intra-Class Correlation. For example, the p value of 0.0084 meant the T-test result for the expression of V3's EOEC-pattern in V1 comparing the EC-EO and EO-EC groups. ICC of 0.46 is the ICC between the expressions of V3's EOEC-pattern in V1 and V2 across all subjects*.

### Comparison between EOEC-pattern and univariate statistical *T* map

**Figure 4:**
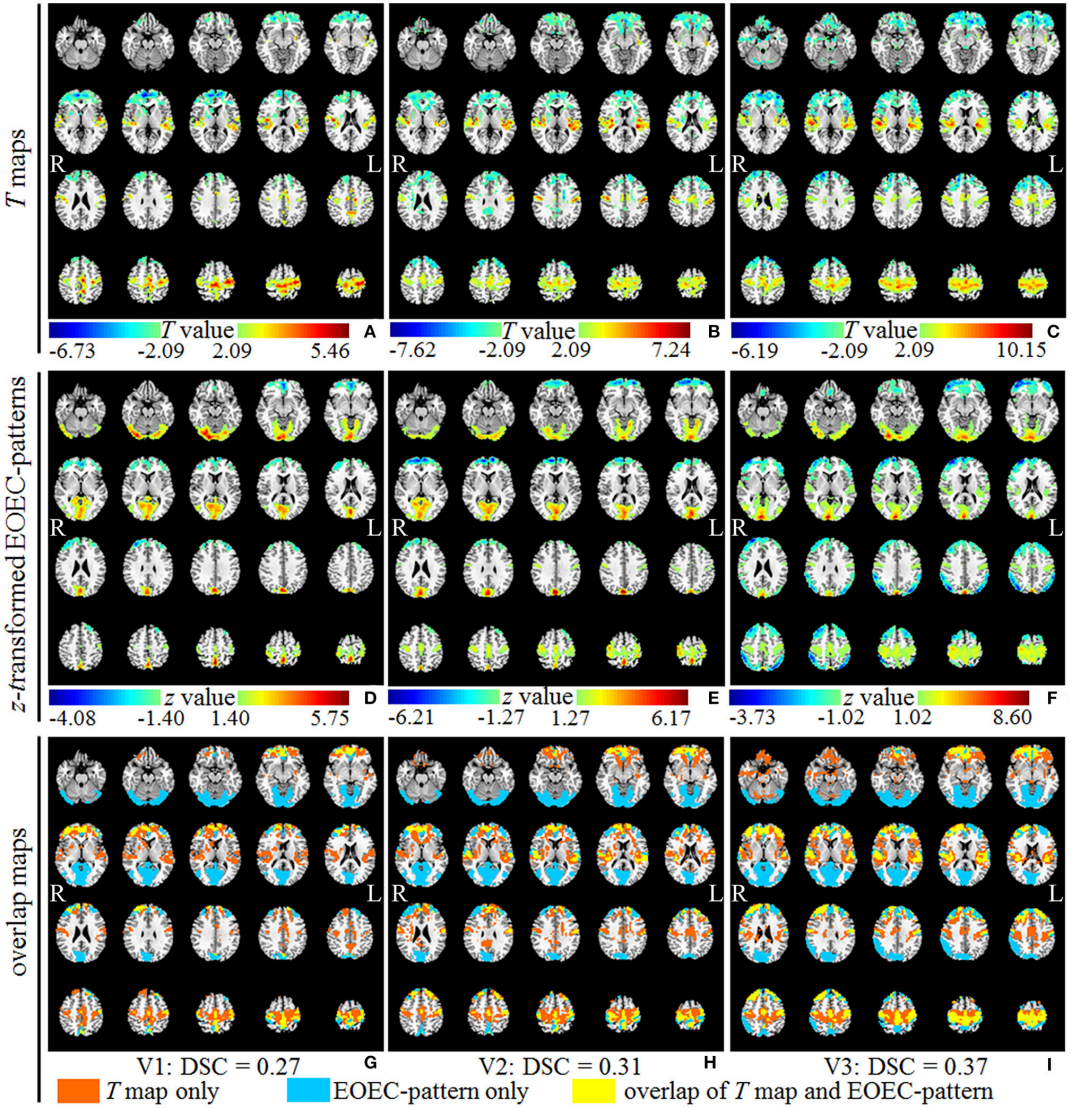
The upper, middle, and bottom rows represented the univariate *T* maps **(A–C)** (with |*T*| > 2.09 and cluster size >915 voxels for V1, 952 for V2, and 1137 for V3), z-transformed EOEC-patterns **(D–F)** (with total number of voxels the same as that of corresponding *T* maps), and overlap maps between *T* maps and EOEC-patterns **(G–I)** of dataset V1, V2, and V3, respectively. The Dice Similarity Coefficient (DSC) for V1, V2, and V3 were 0.27, 0.31, and 0.37, respectively. The *z* coordinates of slices were from −25 to 70 mm with slice spacing of 5 mm.

Figures 4A–C showed the univariate statistical *T* maps between the EC-EO group and EO-EC group with a combined *p* threshold and cluster size threshold described in Table 1. As shown in Figures 4G–I, the DSCs for V1, V2, and V3 were 0.27, 0.31, and 0.37, respectively, suggesting that the EOEC-pattern was quite different from the *T* maps. By visual inspection on Figures 4G–I, *T*-test detected larger, but not exclusively, areas in the primary sensorimotor area and superior temporal gyrus, whereas EOEC-pattern detected exclusively large area in the occipital lobe.

## Discussion

### EOEC-pattern identification

The first pattern, namely EOEC-pattern, accounted substantially more variance than that of the second GIS (GIS2) in each visit (Figure 1 and Supplementary Table 1). The EOEC-pattern included the primary sensorimotor cortex, visual cortex, and frontal cortex (Figures 2A–C). Similar brain areas were also reported in previous EO and EC studies by using paired *T*-tests (Yang et al., 2007; Yan et al., 2009; Liu et al., 2013; Yuan et al., 2014; Zou et al., 2015a). The SSF showed significant differences between the EC-EO group and EO-EC group (Figures 2D–F). Many previous PET studies have consistently found that the first pattern was the disease-related pattern (Ma et al., 2007; Pagani et al., 2016; Tomše et al., 2017). After centralization, the voxel-wise similarity between groups was reduced, while the difference between groups was highlighted (Moeller and Strother, 1991). The current study randomly assigned one group of subjects into EC-EO and EO-EC subgroups, and applied SSM-PCA in the same way as in previous studies. Thus, it is not surprising why the first pattern accounted for the largest portion of total variance, and hence, is the “between-group” difference-related pattern, i.e., EOEC-pattern.

### Intra- and inter-scanner reliability of SSM-PCA

We firstly found high similarity among the EOEC-patterns of the 3 visits. We then calculated the reliability of SSF (i.e., the expression) corresponding to EOEC-patterns. Both the intra- and inter-scanner reliability of SSF was fair to good (ICC = 0.49–0.66) (Figure 3). Furthermore, we calculated the SSF of an EOEC-pattern of one visit onto the other two visits. For example, the EOEC-pattern of V1 was expressed onto V2 and V3, respectively. We found that each EOEC-pattern of one visit could be successfully applied to other two visits to differentiate the EC-EO group and EO-EC group (*p* < 0.01) (Table 3). The ICC value was similar as before (Table 3 vs. Figure 3).

To the best of our knowledge, only one previous study investigated the test-retest reliability of SSM-PCA in two groups of subjects (Ma et al., 2007). Ma and colleagues obtained a Parkinson's disease (PD)-related pattern (PDRP) from a dataset of PD patients and healthy controls. Then this PDRP was expressed onto other datasets to measure the ICC of PDRP' expression (i.e., SSF). They found excellent test-retest reliability over different intervals including 1 h apart (ICC = 0.94 for healthy subjects, and ICC = 0.96 for unmediated PD patients), 1 day apart (ICC = 0.99 for unmedicated early state patients), and 2 months apart (ICC = 0.96 for medicated moderate stage PD patients). These results suggest that the test-retest reliability of FDG PET was higher than that with RS-fMRI ALFF in the present study. This discrepancy might be attributed to differences in the experimental design, imaging modality, as well as computing algorithm of imaging metrics. Simultaneous resting-state PET-fMRI studies have shown that only a small part of brain regions demonstrated significant voxel-level correlation between glucose metabolism and RS-fMRI metrics including ALFF (Aiello et al., 2015; Bernier et al., 2017). Generally speaking, PET and fMRI measure different physiological features. However, the different computation for the two techniques may also account for their discrepancies. The metric for PET glucose metabolism is usually the averaged or integrated value over a period of time, while ALFF of RS-fMRI is the amplitude of fluctuation over time (Zang et al., 2007). A non-invasive perfusion-weighted MRI technique, arterial spin labeling (ASL) is widely used to measure CBF. Some ASL sequences allow calculating both mean CBF over a period of time and CBF-ALFF. A study used ASL and BOLD RS-fMRI to compare between EO and EC states (Zou et al., 2015b). ASL-ALFF and BOLD-ALFF detected similar regions, but CBF-ALFF and CBF-mean detected very different regions. Zou and colleagues also found that CBF-mean showed better test-retest reliability than BOLD-ALFF (Zou et al., 2015a).

### Comparison between EOEC-pattern and univariate statistical *T* map

Both SSM-PCA and univariate *T*-test are statistical methods, however, they are quite different in their mathematical foundations. As a multivariate statistical approach, SSM-PCA obtains patterns and the patterns' expression of each subject based on the covariance matrix of all the voxels from all the subjects, which is a kind of pattern analysis (Alexander and Moeller, 1994; Eidelberg, 2009). The patterns are whole-brain images. Then two-sample *T*-test is applied to the patterns' expression (i.e., SSF) to assess whether the SSF is different between two groups of subjects. If the difference is significant, the corresponding pattern is then named as difference-related pattern, e.g., EOEC-related pattern in the current study or PD-related pattern (PDRP) in previous studies (Ma et al., 2007; Wu et al., 2013, 2015; Tomše et al., 2017). For a voxel in the difference-related pattern, larger value means more contribution or weight to the difference. On the other hand, for univariate statistical method such as voxel-wise *T*-test, comparison is made between the values of each single voxel from two groups of subjects (two-sample *T*-test) or two conditions within a group of subjects (one-sample *T*-test). The total number of comparisons is very different for the SSM-PCA and *T*-test. For SSM-PCA, the total number of comparisons is up to the total number of patterns (up to 21 in the current study), but usually only a few principle patterns are taken into account. And in practice like in the current study and previous studies (Ma et al., 2007; Pagani et al., 2016; Tomše et al., 2017), only the first one component was used because the first component accounts much more variance than the second one. For voxel-wise *T*-test, the total number of comparisons is the total number of voxels (70,831 voxels in the current study). Therefore, false discovery problem due to multiple comparisons is much more severe for univariate statistical method in neuroimaging studies (Poldrack et al., 2011).

The aforementioned points reflected merely a theoretical issue from methodological perspective. From neurophysiological perspectives, SSM-PCA is operating on the notion that localized changes engage multiple, interacting brain regions that are widely distributed over the whole brain owe to intrinsic connectivity in neural substrates. A primary example to support this view is the modulation of SSM*-*PCA pattern and clinical correlation by neurosurgical interventions delivered locally on any key nodes in the pattern (Peng et al., 2014). On the other hand, *T*-test is relying on mean signal in image data to localize regionally- independent group differences over the whole brain. This method does not explicitly account for important functional interactions between different brain regions except neighborhood autocorrelations on a small scale inherent in image data.

We compared the EOEC-pattern with *T*-test pattern. While the results showed overlapped brain regions, they also showed method-specific brain regions. The *T*-test detected larger brain regions in the primary sensorimotor area and superior temporal gyrus, but SSM-PCA detected exclusively large visual area. In a previous research study with voxel-wise paired *T*-test analysis, it was also found that the mean CBF from ASL technique was significantly lower under EC than EO conditions in the primary visual cortex, which was not detected with ALFF *T*-test (Zou et al., 2015a). Moreover, it is well known that the visual cortex can be activated by visual input, so it is reasonable to detect visual area in the EOEC-pattern. Differences that are not significant enough in *T*-test may show up in pattern from SSM-PCA as reported in PET literatures (Habeck et al., 2008; Ma et al., 2009) and ASL literature (Asllani et al., 2008). Consequently, SSM-PCA and univariate *T*-test are two complementary data analytic approaches for application studies.

### Limitations

One limitation is the experimental design for inter-scanner reliability. The current study aimed to investigate both intra- and inter-scanner reliability. When we kept the interval of the two visits of intra-scanner scanning to be similar across subjects, it is impossible to keep the order of inter-scanner scanning count-balanced. Therefore, the second visit was always before the third visit. It means that the reliability between the second and third scanning is a mixed effect of inter-scanner and test-retest reliability. Another limitation is using within-group designs to simulate between-group designs for SSM-PCA.

## Conclusions

Both the intra- and inter-scanner reliability of SSM-PCA of RS-fMRI ALFF was fair to good. The difference-related pattern of SSM-PCA and *T* maps was similar but also showed method-specific brain regions, indicating that the SSM-PCA and *T*-test are two complementary statistical methods.

## Author contributions

Y-FZ, YM, and H-JH conceived and designed the experiment. L-XY, J-BW, and NZ performed the data analysis. L-XY, Y-YL, and D-QL acquired the data. J-BW, YM, and J-HZ provided advices on the analysis and interpretation of the results. L-XY, J-BW, H-JH, YM, and Y-FZ wrote the paper.

### Conflict of interest statement

The authors declare that the research was conducted in the absence of any commercial or financial relationships that could be construed as a potential conflict of interest.
