# Supplementary material for: Intra- and Inter-scanner Reliability of Scaled Subprofile Model of Principal Component Analysis on ALFF in Resting-State fMRI Under Eyes Open and Closed Conditions
Source: Front Neurosci. 2018 May 25;12:311. doi: 10.3389/fnins.2018.00311 (PMC5981094; doi:10.3389/fnins.2018.00311)
Supplement: Supplementary file 1 [file Table_1.docx]

Supplementary Material

Intra- and inter-scanner reliability of scaled subprofile model of principal component analysis in resting-state fMRI

**Li-Xia Yuan^1†^, Jian-Bao Wang^2,3,4†^, Na Zhao^2,3,4^, Yuan-Yuan Li^2,3,4^,Yilong Ma^5*^, Dong-Qiang Liu^6^, Hong-Jian He^1*^, Jian-Hui Zhong^1^, Yu-Feng Zang^2,3,4*^**

*** Correspondence:** yma@northwell.edu (Yilong Ma) or hhezju@zju.edu.cn (Hong-Jian He) or zangyf@gmail.com (Yu-Feng Zang)

# Supplementary Tables

**Supplementary Table 1. The VAF, *p* value, *T* value and Cohen *d* of the whole GISs and SSFs from SSM-PCA in V1, V2, and V3.**

|  |  | GIS1 | GIS2 | GIS3 | GIS4 | GIS5 | GIS6 | GIS7 | GIS8 | GIS9 | GIS10 | GIS11 | GIS12 | GIS13 | GIS14 | GIS15 | GIS16 | GIS17 | GIS18 | GIS19 | GIS20 | GIS21 |
| --- | --- | --- | --- | --- | --- | --- | --- | --- | --- | --- | --- | --- | --- | --- | --- | --- | --- | --- | --- | --- | --- | --- |
| %VAF of V1 | | 30.09 | 10.80 | 7.58 | 6.20 | 5.02 | 4.74 | 3.82 | 3.58 | 3.43 | 3.16 | 2.88 | 2.63 | 2.45 | 2.39 | 2.29 | 2.17 | 2.04 | 1.86 | 1.57 | 1.29 | 0.00 |
| %VAF of V2 | | 25.09 | 12.15 | 7.81 | 7.45 | 5.22 | 5.01 | 4.45 | 3.63 | 3.41 | 3.32 | 3.01 | 2.92 | 2.61 | 2.43 | 2.35 | 2.23 | 1.96 | 1.90 | 1.72 | 1.32 | 0.00 |
| %VAF of V3 | | 31.26 | 8.12 | 6.42 | 5.94 | 5.24 | 4.34 | 4.20 | 3.84 | 3.68 | 3.26 | 2.97 | 2.86 | 2.74 | 2.59 | 2.53 | 2.34 | 2.12 | 2.01 | 1.96 | 1.58 | 0.00 |
|  | | SSF1 | SSF2 | GIS3 | SSF4 | SSF5 | SSF6 | SSF7 | SSF8 | SSF9 | SSF10 | SSF11 | SSF12 | SSF13 | SSF14 | SSF15 | SSF16 | SSF17 | SSF18 | SSF19 | SSF20 | SSF21 |
| *p* of V1 | | 0.012 | 0.61 | 0.11 | 0.38 | 0.11 | 0.68 | 0.44 | 0.58 | 0.32 | 0.60 | 0.62 | 0.56 | 0.74 | 0.24 | 0.18 | 0.84 | 0.40 | 0.76 | 0.43 | 0.87 |  |
| *T-*value of V1 | | 2.77 | 0.52 | -1.67 | -0.89 | 1.66 | -0.42 | -0.80 | 0.57 | -1.02 | -0.53 | -0.50 | -0.60 | -0.34 | 1.21 | -1.39 | 0.20 | -0.86 | -0.31 | 0.81 | 0.17 |  |
| Cohen *d of V1* | | 1.05 | 0.23 | -0.70 | -0.39 | 0.70 | -0.19 | -0.35 | 0.25 | -0.44 | -0.23 | -0.22 | -0.27 | -0.15 | 0.52 | -0.59 | 0.09 | -0.38 | -0.14 | 0.36 | 0.07 |  |
| *p* of V2 | | 0.0044 | 0.10 | 1.00 | 0.40 | 0.96 | 0.05 | 0.33 | 0.79 | 0.71 | 0.94 | 0.45 | 0.74 | 0.60 | 0.31 | 0.47 | 0.64 | 0.29 | 0.94 | 0.75 | 0.66 |  |
| *T-*value of V2 | | 3.23 | 1.72 | -0.01 | -0.87 | -0.04 | -2.12 | 1.00 | 0.27 | 0.38 | 0.07 | 0.77 | -0.34 | -0.54 | 1.04 | -0.73 | -0.47 | 1.09 | -0.08 | 0.32 | 0.45 |  |
| Cohen *d of V2* | | 1.16 | 0.72 | -0.00 | -0.38 | -0.02 | -0.85 | 0.44 | 0.12 | 0.17 | 0.03 | 0.34 | -0.15 | -0.24 | 0.45 | -0.32 | -0.21 | 0.47 | -0.04 | 0.14 | 0.20 |  |
| *p* of V3 | | 0.00062 | 0.13 | 0.16 | 0.48 | 0.25 | 0.38 | 0.89 | 0.37 | 0.93 | 0.49 | 0.65 | 0.48 | 0.79 | 0.82 | 0.85 | 0.93 | 0.93 | 0.32 | 0.80 | 0.76 |  |
| *T-*value of V3 | | 4.09 | -1.57 | 1.46 | -0.72 | -1.19 | -0.91 | -0.14 | -0.92 | 0.09 | -0.70 | -0.46 | 0.72 | -0.27 | -0.23 | 0.19 | -0.09 | -0.09 | 1.02 | 0.26 | 0.31 |  |
| Cohen *d of V3* | | 1.34 | -0.66 | 0.62 | -0.32 | -0.52 | -0.40 | -0.06 | -0.40 | 0.04 | -0.31 | -0.21 | 0.32 | -0.12 | -0.10 | 0.09 | -0.04 | -0.04 | 0.44 | 0.12 | 0.14 |  |

**Supplementary Table 2. The ICC of the SSFs from SSM-PCA in V1, V2, and V3.**

|  | SSF1 | SSF2 | GIS3 | SSF4 | SSF5 | SSF6 | SSF7 | SSF8 | SSF9 | SSF10 | SSF11 | SSF12 | SSF13 | SSF14 | SSF15 | SSF16 | SSF17 | SSF18 | SSF19 | SSF20 | SSF21 |
| --- | --- | --- | --- | --- | --- | --- | --- | --- | --- | --- | --- | --- | --- | --- | --- | --- | --- | --- | --- | --- | --- |
| ICC(V1 vs. V2) | 0.49 | 0.14 | 0.35 | 0.14 | -0.13 | -0.38 | 0.00 | 0.33 | -0.37 | -0.18 | -0.18 | -0.28 | 0.25 | -0.10 | 0.16 | -0.21 | 0.03 | 0.03 | -0.03 | 0.13 |  |
| ICC(V1 vs. V3) | 0.65 | -0.02 | -0.08 | -0.02 | 0.09 | -0.27 | -0.22 | 0.24 | 0.14 | -0.30 | -0.23 | 0.13 | -0.18 | -0.37 | 0.33 | -0.13 | -0.15 | -0.01 | 0.10 | -0.14 |  |
| ICC(V2 vs. V3) | 0.66 | -0.28 | 0.19 | 0.01 | 0.19 | -0.01 | 0.05 | 0.07 | -0.14 | -0.15 | 0.54 | 0.00 | 0.19 | 0.24 | 0.28 | 0.02 | 0.02 | -0.21 | 0.00 | -0.42 |  |
